# Preparation and Electrochemical Properties of Pomegranate-Shaped Fe_2_O_3_/C Anodes for Li-ion Batteries

**DOI:** 10.1186/s11671-018-2757-1

**Published:** 2018-10-30

**Authors:** Zhifeng Wang, Xiaomin Zhang, Yan Zhao, Meixian Li, Taizhe Tan, Minghui Tan, Zeren Zhao, Chengzhi Ke, Chunling Qin, Zhihong Chen, Yichao Wang

**Affiliations:** 1 School of Materials Science and Engineering, Research Institute for Energy Equipment Materials, Hebei University of Technology, Tianjin, 300130 China; 2 Synergy Innovation Institute of GDUT, Heyuan, 517000 Guangdong Province China; 3 Shenyang Institute of Automation, Chinese Academy of Sciences, Guangzhou, 511458 China; 4 School of Life and Environmental Sciences, Deakin University, Waurn Ponds, VIC 3216 Australia

**Keywords:** Li-ion battery, Fe_2_O_3_, Anode, Pomegranate shape, Composite

## Abstract

Due to the severe volume expansion and poor cycle stability, transition metal oxide anode is still not meeting the commercial utilization. We herein demonstrate the synthetic method of core-shell pomegranate-shaped Fe_2_O_3_/C nano-composite via one-step hydrothermal process for the first time. The electrochemical performances were measured as anode material for Li-ion batteries. It exhibits excellent cycling performance, which sustains 705 mAh g^−1^ reversible capacities after 100 cycles at 100 mA g^−1^. The anodes also showed good rate stability with discharge capacities of 480 mAh g^−1^ when cycling at a rate of 2000 mA g^−1^. The excellent Li storage properties can be attributed to the unique core-shell pomegranate structure, which can not only ensure good electrical conductivity for active Fe_2_O_3_, but also accommodate huge volume change during cycles as well as facilitate the fast diffusion of Li ion.

## Background

As a high-performance green chemical power source, lithium-ion batteries (LIBs) have been widely used in portable mobile electronics markets and in electric vehicles due to its high energy density, long cycle life, low self-discharge, and lack of a memory effect [1]. However, with the development of the time, the traditional LIBs based on a graphite material cannot satisfy the growing requirements of high energy density and power density because of the low theoretical capacity (372 mAh g^−1^) of graphite material [2]. Transition metal oxides (TMOs) have been thriving over the past decades with the purpose of achieving superior specific capacities to commercial graphite [3, 4]. Typically, Fe_2_O_3_ has been regarded as one of the most promising anode candidate due to its high theoretical capacity (1007 mAh g^−1^), environmentally friendly nature, non-toxicity, and natural abundance [5, 6]. Despite its tremendous potential, however, its commercial application in LIBs is still hindered by some serious disadvantages such as the fast capacity fading and the volume expansion [7] during discharge/charge process.

To overcome the above issues and improve the electrochemical performance, various optimization strategies have been proposed. A well-accepted strategy [8] is design of nanostructured composite electrode, which not only better accommodates large strains but also provides short diffusion paths for lithium-ion insertion/extraction. To date, lots of nanostructured Fe_2_O_3_ materials including nanoparticles, nanorods, nanowires, and nanotubes have been designed and fabricated by different methods [9–15]. With the help of nanostructure, the volume expansion of Fe_2_O_3_ can be effectively accommodated. Furthermore, TMO-based LIB performance has been further improved by introducing nanostructured TMOs into conductive matrices recently [15–19]. For instance, the introduction of carbon coating layers onto Fe_2_O_3_ core has been widely explored due to the capacity of the carbon layer to enhance electrical conductivity effectively and restrain the cracking and crumbling of the Fe_2_O_3_ anode upon cycling. Zhao et al. [20] prepared Fe_2_O_3_ nanoparticles and graphene oxides through hydrothermal and Hummers’ [21] method respectively. Then, graphene-Fe_2_O_3_ composites were obtained by freeze drying process. Some Fe_2_O_3_–C core-shell composites such as carbon nanotube@Fe_2_O_3_@C, Fe_2_O_3_@C hollow spheres, and Fe_2_O_3_@graphite nanoparticles were fabricated by two-step synthesis methods containing hydrothermal reactions and high-temperature calcination processes [22–24]. These composites have shown excellent Li storage properties. However, the complicated preparation process, long treatment time, and high cost of these composites restrict their further applications. Therefore, developing a simpler approach for Fe_2_O_3_–C core-shell structure is urgently needed.

Herein, we report a synthesis of the Fe_2_O_3_/carbon core-shell nano-composite via a simple one-step hydrothermal process. The resultant Fe_2_O_3_/C nano-composite possesses pomegranate-like structure in which Fe_2_O_3_ was capsuled in carbon shells and every core-shell connects with each other as a pomegranate. This unique porous pomegranate structure can not only ensure good electrical conductivity for active Fe_2_O_3_, but also accommodate huge volume change during cycles as well as facilitate the fast diffusion of Li ion. As a result, the anodes exhibited a remarkable performance improvement when they were used in LIBs.

## Methods

Iron nitrate nonahydrate (Fe_3_(NO_3_)_3_·9H_2_O), anhydrous dextrose (C_6_H_12_O_6_), anhydrous ethanol (CH_3_CH_2_OH), polyvinylidene difluoride (PVDF), and *N*-methyl-2-pyrrolidinone (NMP) were purchased from Tianjin Fuchen Chemical Reagents Factory, China. Deionized water (H_2_O) was provided by Hebei University of Technology.

The pomegranate-shaped Fe_2_O_3_/C nano-composite was prepared by a hydrothermal method. Firstly, 1.212 g Fe_3_(NO_3_)_3_·9H_2_O and 0.9 g C_6_H_12_O_6_ were dissolved in 40 mL of deionized water by magnetic stirring for 30 min, the ratio of carbon in the C_6_H_12_O_6_ to iron in the Fe_3_(NO_3_)_3_·9H_2_O is 10:1. Secondly, the solution was sealed in a capacity of 100 ml Teflon-lined autoclave and heated to 190 °C for 9 h and cooled naturally to room temperature. Then, the hydrothermal synthesis products were taken out and centrifugally separated with deionized water. Last, the products were dried in the thermostatic drying chamber at 60 °C for 12 h.

The phase composition of the samples was investigated by powder XRD on a Rigaku D/Max 2500 V/pc X-ray diffractometer with Cu-Kα radiation (*λ* = 1.5406 Å) with scan range (2*θ*) 20~70° and the scan step of 0.02°. Raman spectra were attained with an Ar-ion laser of 532 nm using the in Via Reflex Raman imaging microscope system. The carbon content of pomegranate-shaped Fe_2_O_3_/C nano-composites was estimated by the thermogravimetric analysis (TGA; TA Instruments, SDTQ600) method [22, 24], which showed weight change after heating up. The weight ratio of carbon was calculated as 45.2 wt%. The morphology of the samples was performed by scanning electron microscopy (SEM) (JEOL JSM-6700F). The microstructure was characterized with a JEOL JEM-2100F transmission electron microscope (TEM), and the elemental composition of the samples was analyzed by energy-dispersive X-ray spectroscopy (EDS). The elements and its valence states were analyzed by X-ray photoelectron spectroscopy (XPS; VG ESCALAB MK II, VG Scientific).

In order to investigate the electrochemical performance, active materials (80 wt%), Super-P (10 wt%), and polyvinylidene fluoride (PVDF, 10 wt%) were mixed in *N*-methyl-2-pyrrolidinone (NMP) to form a slurry. Then, the slurry was coated onto a Cu foil substrate and dried at 100 °C for 6 h. The active materials were used as the working electrode and Li metal foil was used as the counter electrode, 1 mol L^−1^ LiPF_6_ in ethylene carbonate (EC) and dimethyl carbonate (DMC) (1:1 by volume) was used as the electrolyte, Celgard 2300 was used as the separator, and CR2025 coin cells were assembled in an argon-atmosphere glove box. Cycling tests were tested at 25 °C using a CT-4008 battery cycler system between 0.01 and 3.00 V at a current density of 100 mA g^−1^ for 100 cycles. The rate testing at different current densities (10 cycles each at 100 mA g^−1^, 200 mA g^−1^, 500 mA g^−1^, and 2000 mA g^−1^) was followed by an additional cycle test at 100 mA g^−1^. Cyclic voltammetry (CV) was performed on an electrochemical workstation (Zahner Im6e) at a scanning rate of 0.5 mV s^−1^ in a potential range of 0.01~3 V (vs. Li/Li^+^) at room temperature. For comparison, the electrochemical performance of Fe_2_O_3_ nanospheres (25~50 nm, CAS no. 1309-37-1, purchased from Shanghai Aladdin Biochemical Technology Co. Ltd.) was also tested using a same measuring parameter.

## Results and Discussion

**Fig. 1:**
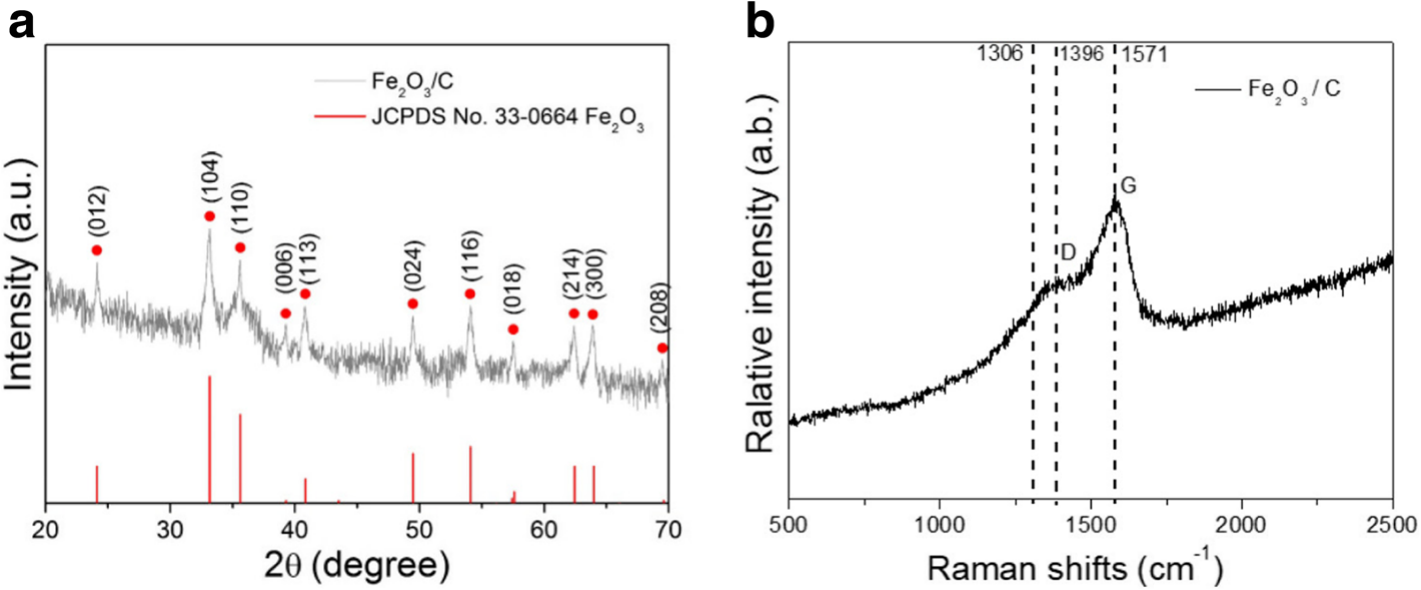
**a** XRD patterns of Fe_2_O_3_/C nano-composite. **b** Raman spectra of Fe_2_O_3_/C nano-composite

The crystallographic structures of Fe_2_O_3_/C nano-composite are confirmed by XRD, and the result is shown in Fig. 1a. It can be seen that XRD pattern of Fe_2_O_3_/C nano-composite could be indexed as the hematite crystal structure of Fe_2_O_3_ (JPDS No. 33-0664). Diffraction peaks of Fe_2_O_3_ in (012), (104), (110), (006), (113), (024), (116), (018), (214), (300), and (208) crystalline plane can be clearly observed. No diffraction peaks of carbon are detected due to the low hydrothermal reaction temperature (190 °C) that is below the crystallization temperature of carbon.

Raman measurement is used to verify the formation of Fe_2_O_3_/C nano-composite. As shown in Fig. 1b, Raman spectra exhibit the peak located around 1306 cm^−1^ associated with the hematite two-magnon scattering that are the feature of Fe_2_O_3_. Due to that Fe_2_O_3_ was coated with carbon, the peak of Fe_2_O_3_ is not obvious [25]. The peaks at 1396 cm^−1^ and 1571 cm^−1^ are characteristic carbon D-band and G-band peaks, respectively. The former corresponds to the disordered carbon, while the later assigns to 2D-graphite. The low value of intensity ratio between D and G bands (ID/IG) implies high relative amount of graphitic carbon and good electrical conductivity of carbon layer, which is beneficial for the conductivity of Fe_2_O_3_/C nano-composite.

**Fig. 2:**
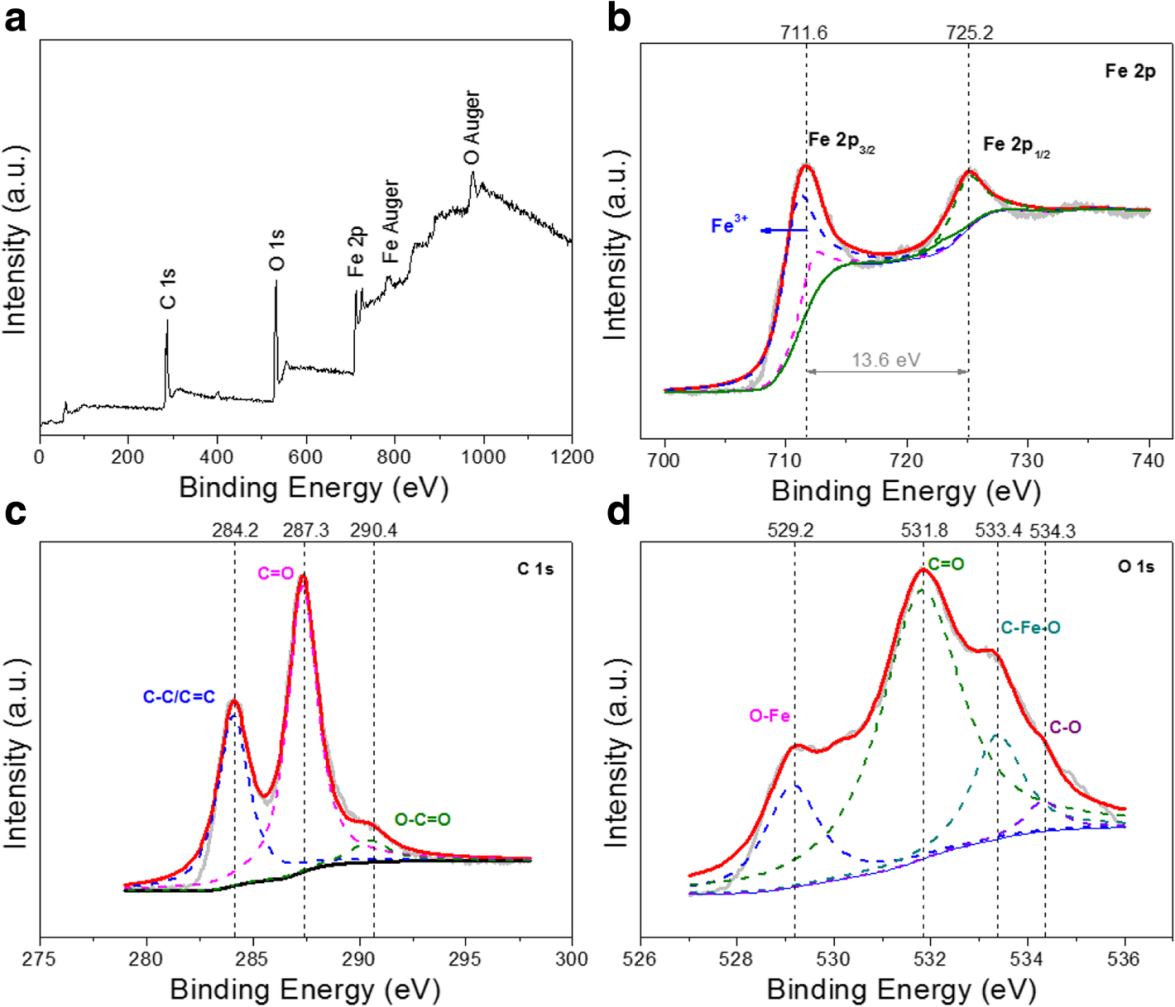
**a** XPS survey spectra of Fe_2_O_3_/C, **b** Fe 2p, **c** C 1s, and **d** O 1s spectra

**Fig. 3:**
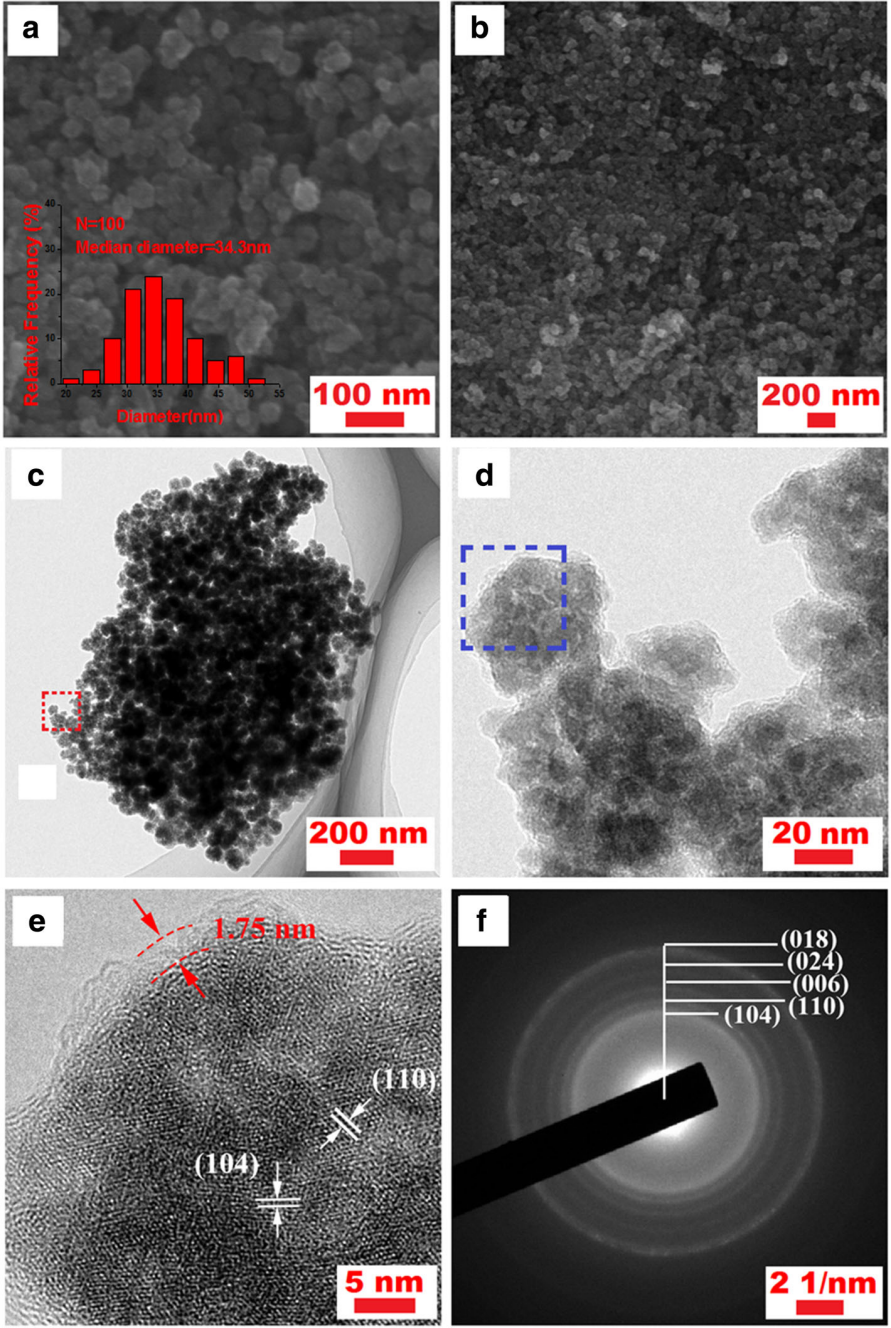
**a**, **b** SEM images of Fe_2_O_3_/C nano-composite; insets: the pore size distribution of Fe_2_O_3_/C composites. **c**, **d** TEM images of Fe_2_O_3_/C nano-composite. **e** High-resolution TEM image and **f** corresponding SAED patterns of Fe_2_O_3_/C

XPS survey spectra are shown in Fig. 2 for further evaluating the chemical compositions and valence states of the product. Figure 2a presents an XPS fully scanned spectra of Fe_2_O_3_/C nano-composite. The C 1s, O 1s, and Fe 2p core photoionization signals and Fe Auger and O Auger signals can be clearly found. An XPS high-resolution scan of the Fe 2p core level is shown in Fig. 2b. It is shown that the peaks at 711.6 and 725.2 eV correspond to Fe 2p_3/2_ and Fe 2p_1/2_ in the Fe 2p spectrum, respectively. The binding energy difference is 13.6 eV which is consistent with the trivalent oxidation state of Fe [26]. The C 1s spectrum of Fe_2_O_3_/C (Fig. 3c) suggests three carbon-containing functional groups: C–C/C=C (284.2 eV), C=O (287.3 eV), and O–C=O (290.4 eV) groups. The presence of the Fe–O–C bond (533.4 eV) in the O 1s spectrum (Fig. 3d) indicates the presence of strong interfacial interactions (Fe–O–C bonds) between Fe_2_O_3_ and carbon-based matrix.

SEM images of Fe_2_O_3_/C nano-composite are shown in Fig. 3a, b. It is clear shown that spherical nanoparticles with uniform size between 30 and 40 nm are homogeneously dispersed. There is lots of space left between particles, forming a 3D conductive structure. The average diameter of the Fe_2_O_3_/C particles was found to be 34.3 nm, as shown in Fig. 3a as inset.

More in-depth information about the Fe_2_O_3_/C nano-composite is further monitored by TEM images (Fig. 3). As shown in Fig. 3c, d, the Fe_2_O_3_ nanoparticles are well-enclosed within carbon shells, implying a pomegranate core-shell structure. According to high-resolution transmission electron microscopy (HRTEM) analysis of Fe_2_O_3_/C core-shell nanoparticle (Fig. 3e), crystalline planes of Fe_2_O_3_ (104), (012) with a distance spacing of 0.33 nm and 0.27 nm can be clearly found, which is in agreement with the above XRD test results. It can also clearly be seen that the Fe_2_O_3_ nanoparticles are well-covered by a carbon layer with a thickness of about 1.75 nm. The corresponding selected area electron diffraction (SAED) pattern confirms that the polycrystalline diffraction ring of the sample corresponds to the Fe_2_O_3_ planes, as shown in Fig. 3f.

**Fig. 4:**
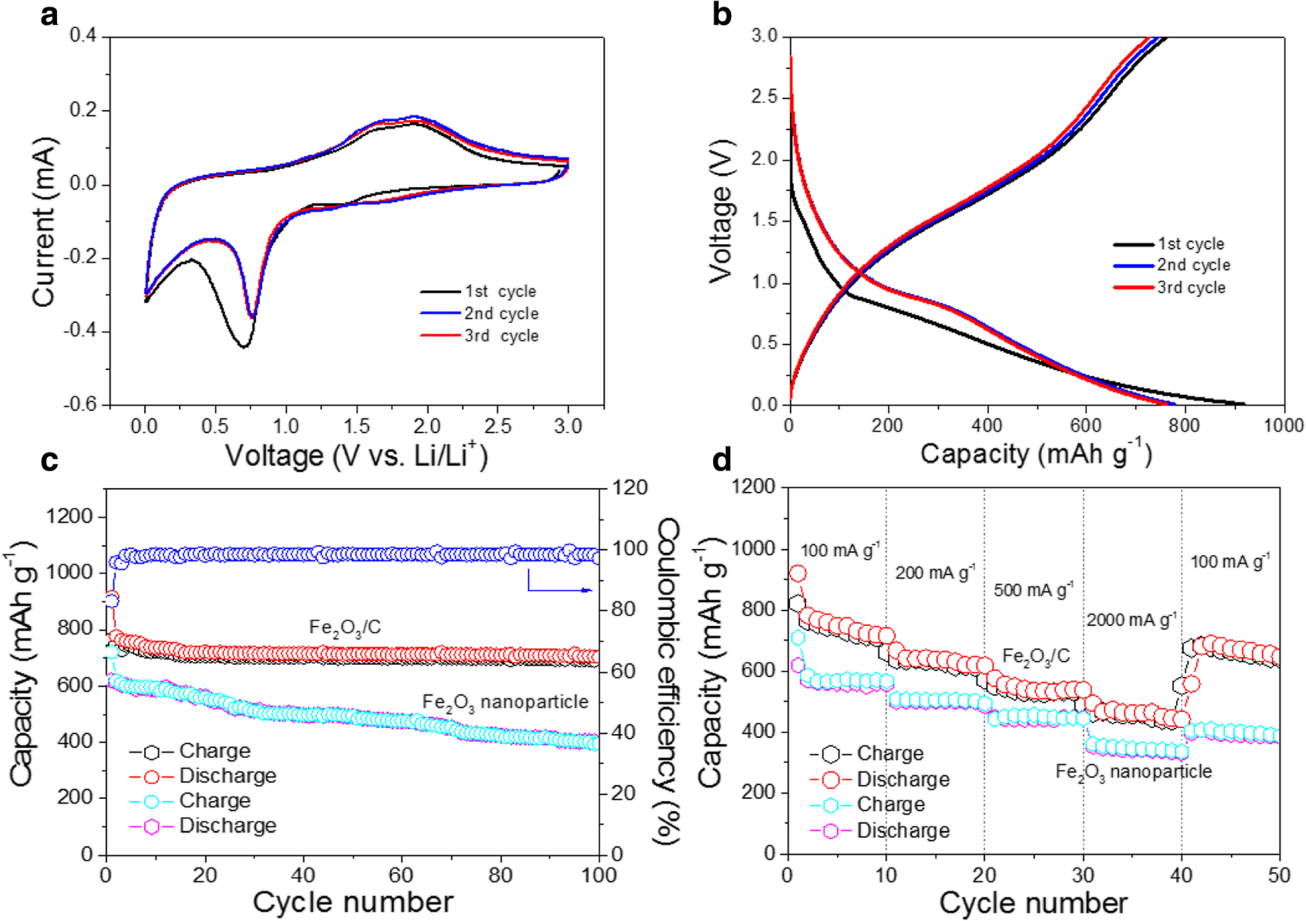
The cyclic voltammogram (**a**) and voltage profiles (**b**) of the Fe_2_O_3_/C composite at the first, second, and third cycle. **c** Cycle performance of Fe_2_O_3_/C and Fe_2_O_3_ nanoparticles at 100 mA g^−1^. **d** Rate capability of Fe_2_O_3_/C and Fe_2_O_3_ nanoparticles with a current density ranging from 100 to 2000 mA g^−1^

Figure 4a depicts the CV plots with a voltage range between 0.01 and 3.0 V at a scan rate of 0.1 mV s^−1^. In the first cycle, the cathodic peak at about 0.7 V was believed to the conversation of Fe^3+^ to Fe^0^ as well as the formation of solid electrolyte interphase (SEI) film, while the broad peak near 0.1 V may be related to the Li^+^ ion insertion in carbon and the formation of LiC_6_ [27]. A dominant anodic peak at 1.75 V can be attributed to the oxidation of Fe^0^ to Fe^3+^. The related reaction can be described by the Eq. (1) [27]:

1 $$ {\mathrm{Fe}}_2{\mathrm{O}}_3+6{\mathrm{Li}}^{+}+6{\mathrm{e}}^{\hbox{-}}\leftrightarrow 2\mathrm{Fe}+3{\mathrm{Li}}_2\mathrm{O} $$

In the following cycles, both positions of cathodic and anodic peaks shifted to a higher potential (0.8 and 1.78 V respectively), which can be ascribed to the improved kinetics of the Fe_2_O_3_ electrode after the structure realignment and electrochemical activation. Meanwhile, the intensities of CV curves dropped slightly, which may resulted from a better electrical contact between electrodes with electrolyte and the formation of a stable SEI film. In addition, the overlapped CV curves in the following cycles implied a good electrochemical reversibility.

The initial three charge/discharge cycling results of Fe_2_O_3_/C electrodes at a constant current density of 100 mA g^−1^ are shown in Fig. 4b. The first discharge capacity of Fe_2_O_3_/C was 917 mAh g^−1^ and was only 760 mAh g^−1^ during charging. The loss of capacity may be caused by the inevitable formation of solid electrolyte interphase (SEI) film. The reversible capacity of the second and third cycle is 776 and 763 mAh g^−1^ respectively. It exhibits the excellent cyclic stability.

The cycling performance of the electrode at a current density of 100 mA g^−1^ is shown in Fig. 4c. The second discharge capacity of the Fe_2_O_3_/C is 776 mAh g^−1^, and after 100 cycles, the electrode retained a specific capacity of 705 mAh g^−1^, which is about 90% of the second discharge capacity, indicating a good cycling performance. And the coulombic efficiency is almost 100% after 100 cycles, further confirming the superior electrochemical performance. The rate performance of the Fe_2_O_3_/C at current density ranging from 100 to 2000 mA g^−1^ is displayed in Fig. 4d. It showed good rate capability, with a charging capacity of 710 mAh g^−1^, 620 mAh g^−1^, 580 mAh g^−1^, and 480 mAh g^−1^ at 100 mA g^−1^, 200 mA g^−1^, 500 mA g^−1^, and 2000 mA g^−1^, respectively. When the rate was returned to 100 mA g^−1^, the capacity of the electrode was back to 680 mAh g^−1^, which showed excellent rate capability. The excellent electrochemical performance is mainly attributed to the enhanced core-shell structural stability, and carbon improves the electric conductivity. Every core-shell structural connects as pomegranate which also can improve electron transfer to improve the electric conductivity and enhance structural stability.

Figure 4c, d also shows the cycling performance of the Fe_2_O_3_ nanoparticle anode at 100 mA g^−1^. The first discharge capacity of the Fe_2_O_3_ nanoparticles is about 720.9 mAh g^−1^, but after 100 cycles, it only retained a specific capacity of 396.5 mAh g^−1^. And the rate performance of the Fe_2_O_3_ nanoparticles at current rates ranging from 100 to 2000 mA g^−1^ is shown in Fig. 4d. The capacity of the Fe_2_O_3_ anode is 570 mAh g^−1^, 505 mAh g^−1^, 450 mAh g^−1^, and 345 mAh g^−1^ at 100 mA g^−1^, 200 mA g^−1^, 500 mA g^−1^, and 2000 mA g^−1^, respectively. When the rate was returned to 100 mA g^−1^, the capacity of the electrode was back to 395 mAh g^−1^. Therefore, the electrochemical rate and cycling performance of Fe_2_O_3_ nanoparticle anode is not a 60% as good as Fe_2_O_3_/C anode, which is mainly because of the volume expansion of Fe_2_O_3_ nanoparticles during the charge and discharge process.

The theoretical capacity (*C*_theo._) of the as-obtained pomegranate-shaped Fe_2_O_3_/C anode is *C*_theo._ = *C*_Fe2O3,theo._ × Fe_2_O_3_% + *C*_carbon,theo._ × Carbon% = 1007 × 54.8% + 372 × 45.2% = 720 mAh g^−1^. After charge/discharge cycling at 100 mA g^−1^ for 100 cycles, the discharge capacity remained at about 705 mAh g^−1^, which is slightly lower than the theoretical capacity. These high capacities may result from the synergistic interactions between Fe_2_O_3_ and carbon.

**Fig. 5:**
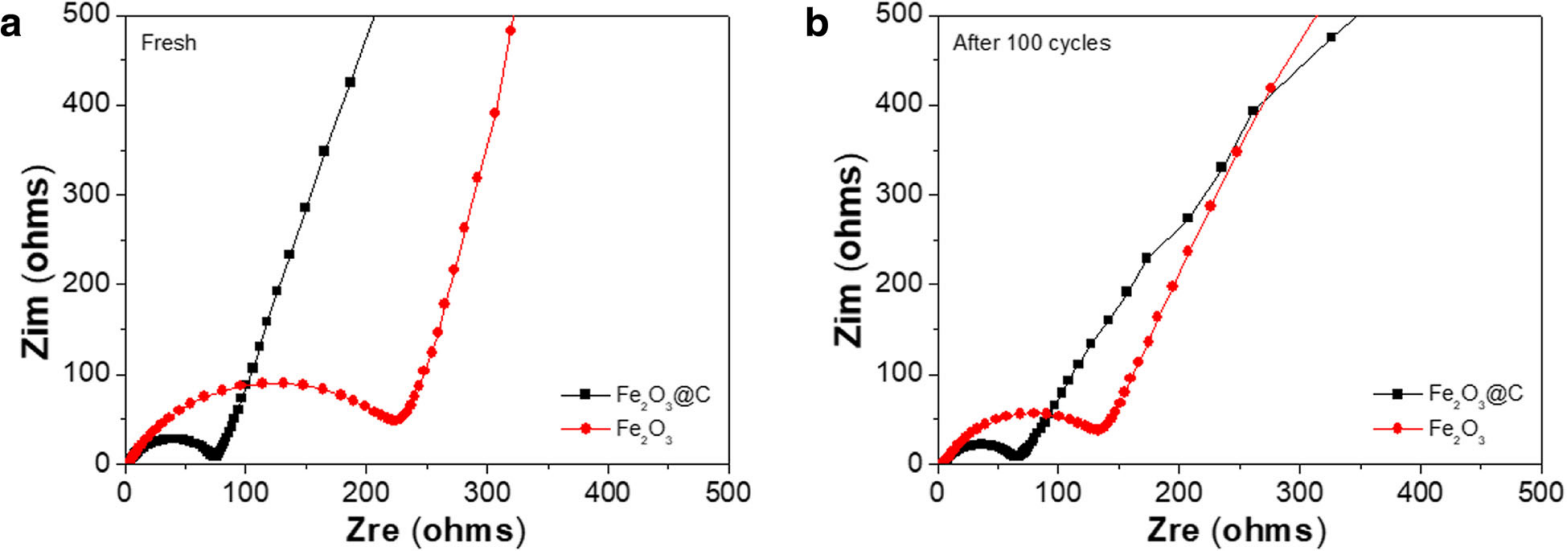
Nyquist plots of Fe_2_O_3_ and Fe_2_O_3_/C electrodes

Figure 5 shows the electrochemical impendence spectroscopy (EIS) of the Fe_2_O_3_ and Fe_2_O_3_/C electrodes before and after 100 cycles. The high-frequency semicircle in the Nyquist plot is connected with the charge transfer resistance of the electrode, whereas the slope line at the low frequency is an indication of Warburg impedance of Li ion into active material diffusion. It is well known that smaller semicircle represents a lower charge transfer resistance of an electrode. Obviously, the diameter of the semicircle for the core-shell pomegranate-shaped Fe_2_O_3_/C composite before and after cycles is much smaller than that of the Fe_2_O_3_ contrast material in the corresponding state, indicating the fact that the core-shell pomegranate-shaped Fe_2_O_3_/C composite electrode possesses lower contact and charge transfer impedances when used as anode materials than the bare Fe_2_O_3_ sample. This result can be attributed to the porous pomegranate-shaped structure of the Fe_2_O_3_/C anode, which can provide more space to adapt the change of volume and promote Li^+^ ion diffusion during lithiation and delithiation processes.

Li storage performances of the as-obtained core-shell pomegranate-shaped Fe_2_O_3_/C anode and related Fe_2_O_3_/C materials reported in the previous literature are summarized in Table 1 [27–34]. It can be seen from the table that the pomegranate-shaped Fe_2_O_3_/C anode shows higher capacity after cycling than most of reported anodes. The excellent performance of the material in Li-ion storage can be attributed to the unique structure of macroscopical pomegranate shape with plentiful porosity as well as microscopic core-shell Fe_2_O_3_–C structure, which can ensure good electrical conductivity for active Fe_2_O_3_, accommodate huge volume change during cycles, and facilitate the fast diffusion of Li ion.

**Table 1:** The comparison of Li storage performances between this work and the previous literature

Anode material	Current density (mA g^−1^)	Cycle number	Reversible capacity (mAh g^−1^)	Reference
Fe_2_O_3_@mesoporous carbon	100	50	703	[28]
Porous Fe_2_O_3_/carbon nanorods	200	100	581	[29]
Fe_2_O_3_–carbon fiber	50	150	634	[30]
Porous Fe_2_O_3_–C microcubes	100	100	516	[31]
Fe_2_O_3_ nanotubes@graphene	100	100	656	[32]
α-Fe_2_O_3_@carbon aerogel	100	50	582	[33]
α-Fe_2_O_3_@graphene aerogel	100	100	745	[27]
Fe_2_O_3_/natural graphite	72	100	687.6	[34]
Core-shell pomegranate-shaped Fe_2_O_3_/C	100	100	705	This work

## Conclusions

In summary, we have successfully designed and synthesized pomegranate-shaped Fe_2_O_3_/C to realize industrialization. The Fe_2_O_3_ nanoparticles are well-enclosed within carbon shells, and every core-shell structure is connected to each other as pomegranate, which not only improves the stability of the anode during discharging/charging process but also leads to the improvement of the lithium reaction kinetics. This structure greatly reduces the volume expansion and provides good electrolyte diffusion. So the Fe_2_O_3_/C composites as the anode of LIB exhibit superior lithium ion storage performance.

## Abbreviations

TMOs: Transition metal oxides
Fe_2_O_3_/C: Fe_2_O_3_/carbon
Fe_3_(NO_3_)_3_·9H_2_O: Iron nitrate nonahydrate
C_6_H_12_O_6_: Anhydrous dextrose
CH_3_CH_2_OH: Anhydrous ethanol
PVDF: Polyvinylidene difluoride
NMP: *N*-Methyl-2-pyrrolidinone
LIBs: Lithium-ion batteries
XRD: X-ray diffraction
SEM: Scanning electron microscope
TEM: Transmission electron microscope
HRTEM: High resolution transmission electron microscopy
EDS: Energy-dispersive X-ray spectroscopy
XPS: X-ray photoelectron spectroscopy
EC: Ethylene carbonate
DMC: Dimethyl carbonate
CV: Cyclic voltammetry
SAED: Selected area electron diffraction
SEI: Solid electrolyte interphase
EIS: Electrochemical impendence spectroscopy.
